# Oral mucositis. Is it present in the immunotherapy of the immune checkpoint pd1/pd-l1 against oral cancer? A systematic review

**DOI:** 10.4317/medoral.24353

**Published:** 2021-03-27

**Authors:** Juan Francisco Peña-Cardelles, Angel Orión Salgado-Peralvo, Pablo Garrido-Martínez, José Luis Cebrián-Carretero, José Juan Pozo-Kreilinger, José Ernesto Moro-Rodríguez

**Affiliations:** 1 Professor of the Postgraduate Program in Oral Surgery and Implantology. Universidad Rey Juan Carlos, Madrid, Spain; 2 Associate Professor. Master in Family and Community Dentistry. Master in Oral Implantology, Faculty of Dentistry, University of Seville, Spain; 3 Associate Professor. Department of Prosthesis, Faculty of Dentistry, University Alfonso X el Sabio, Madrid, Spain; 4 Department of Oral and Maxillofacial Surgery, Hospital La Luz, Madrid, Spain; 5 Chief. Department of Oral and Maxillofacial Surgery, Hospital La Luz, Madrid, Spain; 6 Chief of Section. Department of Oral and Maxillofacial Surgery, Hospital Universitario La Paz, Madrid, Spain; 7 Associate Professor of Medicine. Department of Pathology. Universidad Autónoma de Madrid, Spain; 8 Hospital Universitario La Paz, Madrid, Spain; 9 University Professor. Pathological Anatomy Area, Universidad Rey Juan Carlos, Madrid, Spain

## Abstract

**Background:**

Oral mucositis (OM) is a painful lesion that takes place in the mucosa of the oral cavity, usually its etiology is associated with drug therapies in cancer patients. It is presented as well-defined ulcers whose painful symptomatology sometimes implies the suspension of oncological treatment or parenteral feeding, being therefore an important adverse effect, marking the evolution of these types of therapies against cancer. The present work aim is to know the prevalence of oral mucositis in oral cancer immunotherapy compared to its prevalence in standard therapy.

**Material and Methods:**

A protocol was developed for a systematic review following PRISMA® guidelines and a focused question (PICO) was constructed. A comprehensive literature search was conducted on electronic databases including PubMed, the SCOPUS database, the Cochrane library and the Web of Science (WOS).

**Results:**

Six clinical trials were included that met the different inclusion criteria. In these articles, a discrepancy between the prevalence of OM in patients treated with chemotherapy and patients treated with immunotherapy related to the immune checkpoint PD-1/PD-L1 (Nivolumab and Pembrolizumab) was observed.

**Conclusions:**

The prevalence of oral mucositis is lower in new immunotherapy with monoclonal antibodies against oral cancer than drugs used so far (chemotherapy drugs [methotrexate, cisplatin] as well as cetuximab). However, more studies should be carried out to confirm these data.

** Key words:**Oral mucositis, PD1, PD-L1, oral cancer, immunotherapy.

## Introduction

Cancer represents a disease with a high mortality rate, corresponding to one of the leading causes of death worldwide (1). Oral squamous cell carcinoma (OSCC) is the sixth most common cancer worldwide with a mortality rate of 40-50% (2).

Most patients with OSCC have a poor prognosis due to the late diagnosis of the disease, in which surgical or pharmacological treatment with chemotherapy is limited and with negative results, with a life expectancy of 5 years in 50% of the cases after diagnosis. Besides, unfortunately, the average survival of patients with disease recurrence or metastasis is around 8-10 months (1-4).

Recent studies indicate that immunotherapy is amongst the most promising strategies in modern cancer therapy. It aims to strengthen innate and adaptive immunity, highly specific for tumor cells, and with low toxicity to healthy host cells (4-6).

Cancer triggers an innate and adaptive immune response of the host during its evolution. Immune tolerance to the tumor is recognized as one of the main characteristics of cancer (4). Cancer immunotherapy has been a paradigm shift. Currently, studies on different neoplasms are focused on the development of drugs to modulate the immune system (IS) against the tumor, on identifying the molecules expressed by the tumor and the impact of these molecules against the IS. As a result, studies with enormous perspectives on immunotherapy and the expression of immunoregulatory molecules by neoplasms, have emerged. The immunological checkpoint that has represented a great advance in the knowledge of molecular oncology and immunotherapy has been the PD1 ("Programmed cell death protein 1") and PD-L1 (“Programmed death-ligand 1). The study of the expression of PD1 and PD-L1, as well as clinical trials with drugs involved in their regulation, are the most relevant studies in the field of oncology today (5-7).

The expression of PD1 and PD-L1 in neoplasms has been shown to be relevant in clinical trials with monoclonal antibodies used for their treatment. In head and neck cancer, the development of clinical trials to understand the effect of immunotherapy on the tumor is thriving, and currently, two monoclonal antibodies have been approved for the treatment of this cancer (Nivolumab and Pembrolizumab) that have been used in studies of patients with advanced, recurrent disease and resistant to chemotherapy, allowing their comparative study (4-6).

- Standard therapy and chemotherapy

For oral cancer, the first-line treatment modality is surgery. Unlike other types of cancer where adjuvant treatment such as chemotherapy is given after surgery, this does not take place in the case of oral cancer and chemotherapy is reserved for more advanced carcinomas, depending on their location, size and risk of recurrence (6,7).

The two primary modalities with curative potential in the OSCC are surgery and radiotherapy. In the case of chemotherapy, no promising results have been obtained and its use is mainly as palliative treatment (6,7). Amongst the chemotherapy drugs used we find paclitaxel, docetaxel, methotrexate, 5-fluorouracil, bleomycin and cisplatin (6-9).

A more recent drug in standard therapy, used in combination with radiotherapy, is cetuximab, a monoclonal antibody directed against the epidermal growth factor receptor, which has an established role in the primary treatment of advanced and unfavorably located oral cancer (10,11). However, as noted above, the monoclonal antibodies that are checkpoint inhibitors of PD1 and PD-L1 are achieving superior results compared to therapies used to date (4).

- Oral Mucositis

Cancer treatment usually produces a series of changes on normal cells. The gastrointestinal mucosa, especially the oral mucosa, are very susceptible to both direct and indirect toxic effects from chemotherapy and radiotherapy. In the oral cavity, this risk is the result of several factors, such as the high rate of mucosal cell renewal, the existence of a complex and diverse microflora, and trauma to oral tissues during normal oral function (12-15).

Mucositis is the painful inflammation and ulceration of the mucous membranes that line the digestive tract, commonly developing secondary to cancer treatment with radiotherapy or chemotherapy. Mucositis can occur at any level in the gastrointestinal tract, although it is more common in the mouth, receiving the name of oral mucositis (OM) (14-16).

The World Health Organization (WHO) developed a classification of mucositis, being the mildest form the one that has an erythema with non-specific discomfort, burning sensation and hypersensitivity to food (Grade 1). Grade 2 also causes extensive ulcers and mild pain, and food may still be swallowed. Grade 3 increases the severity of the ulcers and therefore the pain, there is difficulty in speaking and the possibility of intake is limited to liquids. The final grade in this classification, grade 4, is characterized by very extensive ulcers, hyposialia, and very significant pain that makes swallowing and even fluid intake impossible (17,18).

The intensity of the antineoplastic treatment conditions, to a great extent, the appearance of the oral mucosa's adverse effects. The occurrence of OM can be as high as 100% in patients with chemotherapeutic treatments and with radiation in the head and neck region. Patients also report that this is the most uncomforTable side effect they experience from antineoplastic treatment (13).

The present paper aims is to determine the prevalence of oral mucositis in immunotherapy of the immune checkpoint PD1/PD-L1 against cancer compared to its prevalence in standard therapy.

## Material and Methods

A protocol was developed to conduct a systematic review following the PRISMA® Statement guidelines (Preferred Reporting Items for Systematic Reviews and Meta-analysis) (19,20). Based on these guidelines, a focused question “PICO” (*P*=patient/problem/population; I=intervention; C=comparison; O=outcome) was constructed, taking into account different headings corresponding to the study population, the intervention, the comparison and the outcome. In this case, the question asked is the following:

“Is the prevalence of OM in patients with head and neck cancer treated with immunotherapy lower compared to the prevalence of OM in those treated with chemotherapy drugs?”

- Inclusion criteria

Types of studies: (a) Original clinical trial-type articles published in scientific journals; (b) studies carried out on human beings; and (c) studies in the English language.

Type of population: Patients undergoing treatment with FDA-approved immunotherapy drugs for the treatment of head and neck cancer (Pembrolizumab, Nivolumab) or undergoing approval currently being evaluated in clinical trials such as the monoclonal antibody Durvalumab. Studies should reflect adverse effects and, if possible, include a placebo group or be compared with a group with standard therapy used in head and neck cancer.

- Exclusion criteria

Excluded studies have the following characteristics: (a) Those studies that did not reflect the prevalence of mucositis in the compared groups; (b) Those articles whose study base focused on other neoplasms did not include the head and neck region; (c) literature review studies, case reports, letters to the editor, abstracts or conference papers; and (d) Those published in a non-English language.

No time period was set for inclusion and exclusion criteria as the less recent articles were published in 2015.

- Sources of information used

A comprehensive search of the literature was conducted in electronic databases, including PubMed (U.S. National Library of Medicine, National Institutes of Health), the SCOPUS database, the Cochrane library, and the Web of Science (WOS) on April 21, 2019.

- Search Strategy

The search strategy was carried out using the following terms linked through the following Boolean algorithms: "Head and Neck Neoplasms" AND (Pembrolizumab OR Nivolumab OR Durvalumab).

- Bias risk assessment

Bias risk assessment was assessed independently by two reviewers. Disagreements were resolved by consensus between the two reviewers or the intervention of a third author.

Using the predetermined 10 domains for the methodological quality assessment according to the Joanna Briggs Institute Prevalence Critical Appraisal Tool (17), we determined that the papers (24-26) to have a low quality assessment (0–5 domains) and three of them (21-23) to have a high quality assessment (5-10 domains). Table 1 shows a more detailed description of the articles included.

## Results

**Figure 1:**
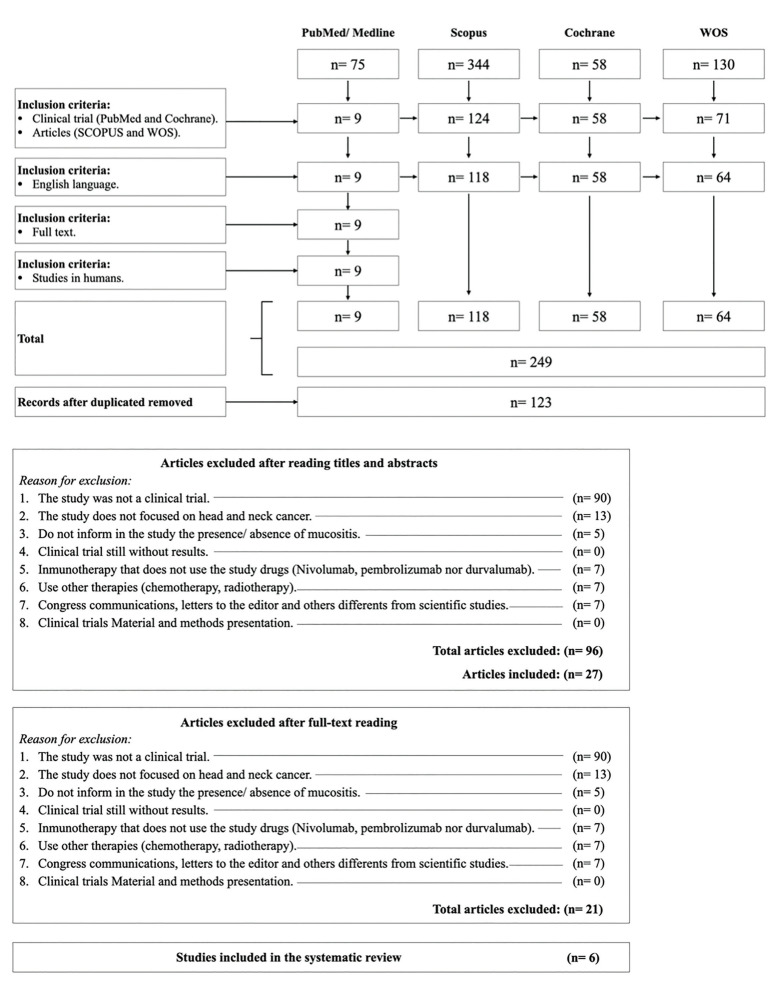
PRISMA flow diagram of the search processes and results.

The initial search showed a total of 607 articles (75 from the PubMed database, 344 from SCOPUS, 58 from the Cochrane Library and 130 from WOS). After applying the first inclusion criterion of clinical trials in PubMed and the inclusion of scientific articles in the rest of the database, the result was reduced to 262 articles (9 from the PubMed database, 124 from SCOPUS, 58 in the Cochrane Library and 71 in WOS) (Fig. 1).

After the application of the other inclusion criteria: English language, full-text studies and studies carried out on human subjects, a total of 249 articles were obtained for consultation (9 in the PubMed database, 118 in SCOPUS, 58 in the Cochrane Library and 64 in WOS). By eliminating the duplicated articles from the different databases, a total of 123 articles remained to be read, since 126 were duplicates. The title and abstract of the 123 articles were read and the flowchart for possible inclusion or exclusion was established. The reasons for exclusion of a total of 96 articles after reading the title and summary can be seen in the appendix to this document. A total of 27 articles were read entirely to determine the articles' final inclusion in this review. In this case, 21 articles were excluded for the reasons reflected in the appendix to this document and in the flow chart, thus only 6 articles were included for the elaboration of the results of this work.

**Table 1:**
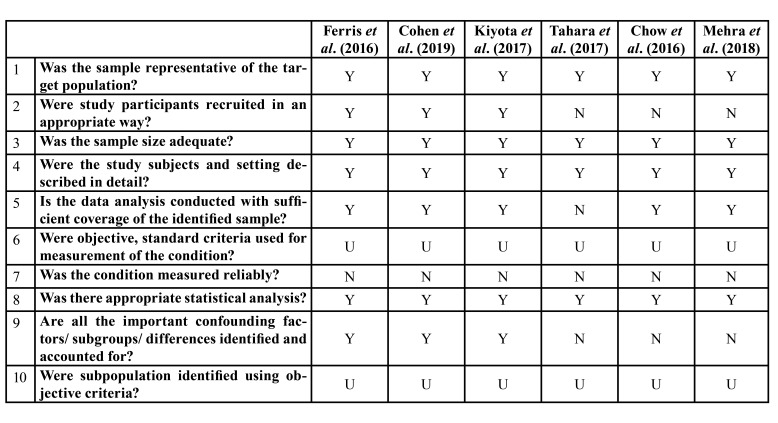
JBI Critical Appraisal Checklist for studies reporting prevalence data.

The data of these 6 articles are summarized in Table 2 and described below:

Ferris *et al*. [2016] present an analysis of the CheckMate 141 clinical trial, a randomized phase III trial in which Nivolumab is studied against the investigator's chosen therapy (methotrexate, docetaxel, or cetuximab) in patients with recurrent metastatic and platinum-resistant head and neck carcinoma after 6 months of treatment (21).

The trial was conducted in 360 patients, with a total of 240 individuals receiving treatment with Nivolumab at a dose of 3 mg/kg every two weeks, while 120 received the investigator's standard treatment. The median survival of the nivolumab group was 7.5 months compared to 5.1 months for the group receiving standard therapy (21).

Total adverse events occurred in 139 patients (58.9%), of which 13.1% of the total sample were grade 3 or 4 adverse events that occurred in the nivolumab group, but these grade 3 or 4 adverse events occurred in 35.1% in the standard therapy group (21).

Data on the presence of OM were reflected in this study, corresponding to 5 cases (2.1%) in the nivolumab group and 10 cases (9%) in the standard therapy group (21).

Cohen *et al*. [2019] present a phase III clinical trial and in their article, they reflect the efficacy and safety of the drug pembrolizumab compared to the standard therapy. This trial involved 97 medical centers in 20 countries. The patients included in the trial were platinum-resistant subjects who did not respond to therapy after 6 months of treatment. Two groups were used, a pembrolizumab group at 200 mg dose every 3 weeks intravenously compared to a group with a standard therapy chosen by the investigator (methotrexate [n=65], docetaxel or cetuximab) (22).

A total of 247 patients received pembrolizumab and 248 received the standard therapy (65 methotrexate, 110 docetaxel, 73 cetuximab). The mean age in the pembrolizumab group was 60 years [55-66], compared to the standard group which was 60 years (range from 54 to 66 years) (22).

The overall survival of the treated population was 8.4 months in the pembrolizumab compared to 6.9 months in the standard group. There were fewer adverse effects with pembrolizumab in comparison to the standard therapy, finding 33 cases with pembrolizumab (13%) versus 85 cases (36%) in the standard (22).

The authors indicate that the most common adverse effect with pembrolizumab is the development of hypothyroidism in 33 patients (n=33; 13%). The most common adverse effect in standard therapy is fatigue in 43 patients (n=43; 18%) (22).

In this article the authors report a prevalence of OM in 6 cases (2%) in the pembrolizumab group and a case of mucositis of a higher grade than 3. While in the standard therapy group, 28 patients had OM (12%) and in 11 of them (5%), it was of a higher grade than 3 (22).

Kiyota *et al*. [2017] analysed the sample of Asian subjects from the CheckMate 141 clinical trial. This sample has a total of 34 patients, 23 in the nivolumab group and 11 patients in the standard therapy group (8 with methotrexate, 2 with docetaxel, 1 with cetuximab) (23).

Adverse effects occurred in 26 subjects, 16 of the nivolumab group (69.6%) and 10 of the standard therapy group (90.9%). Of these adverse effects, grade III or greater occurred in 2 patients in the nivolumab group (8.7%) and 3 in standard therapy (27.3%) (23).

**Table 2:**
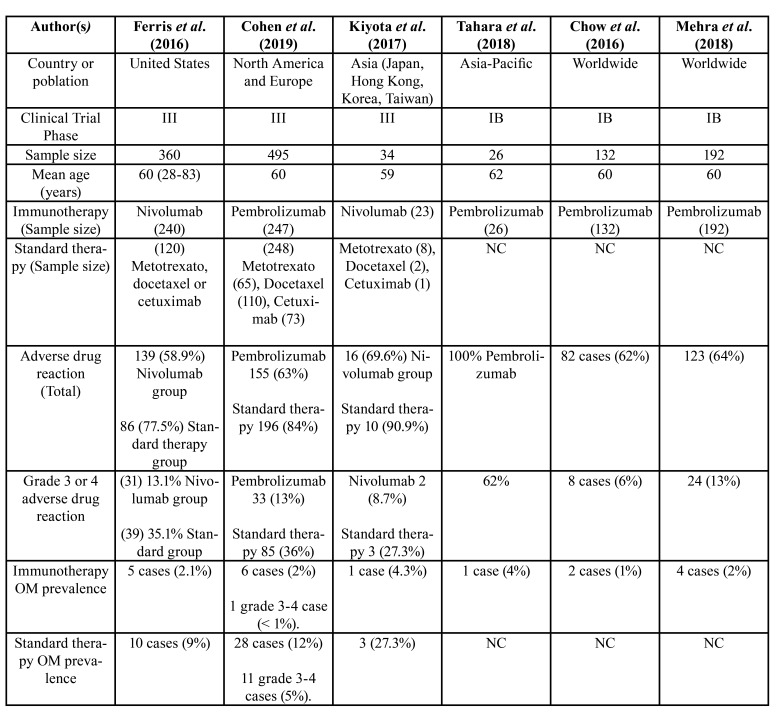
Breakdown of selected studies (NC., not compared; OM., oral mucositis).

Regarding the presence of OM, in the nivolumab group it occurred in 1 case (4.3%), while in the standard therapy it occurred in 3 cases (27.3%) (23).

Tahara *et al*. [2017] is the KEYNOTE-012 clinical trial, in phase I, so there is no control group sample. The drug studied in this trial is Pembrolizumab at a dose of 300 mg every 3 weeks. Twenty-six patients with an average age of 62 years participated and, in this study, 62% of the patients presented adverse effects, finding 1 case (4%) of OM (24).

Chow *et al*. [2016] show the results of the KEYNOTE-012 clinical trial in phase I-B. In this trial they used pembrolizumab at a dose of 200 mg intravenously for 3 weeks in a total of 132 subjects with an average age of 60 years. A total of 82 cases (62%) presented adverse effects, finding a total of 8 cases (6%) with a grade greater than 3 and a prevalence of OM in 2 cases (25).

Mehra *et al*. [2018] also show the results of the long-term KEYNOTE-012 clinical trial. In this analysis a total of 123 adverse effects were obtained (64%), 24 (13%) with a degree greater than 3 and a total of 4 cases of OM (2%) (26). In this trial, the sample of study was 192 patients as opposed to the sample of 132 studied by Chow *et al*.

## Discussion

In order to answer the objective of this work, the review was carried out with special attention to the clinical trials published to date. In these articles a discrepancy between the prevalence of OM in patients treated with chemotherapy and patients treated with immunotherapy (Nivolumab and Pembrolizumab) has been observed.

There is a large discrepancy between the sample size of the different articles. This is due to the fact that some trials are in phase III (21-23), while other trials are in the initial stages and therefore have a smaller number of subjects (24-26). In the case of Kiyota *et al*. (23), despite being a phase III clinical trial, the sample is smaller since the study published by these authors refers to the Asian population only.

The patients' average age was very similar in all the clinical trials included, with the average age being between 59 and 62 years (21-26).

In the phase III clinical trials, the sample to be compared included patients treated with a therapy according to the researcher. This standard therapy was similarly executed in the three phase clinical trials included in this review, with methotrexate, docetaxel or cetuximab being used as therapy (21-23).

The trial by Ferris *et al*. (21) reported a prevalence of OM in the immunotherapy group (nivolumab) of 5 cases (2.1%) compared to 10 cases (9%) in the standard therapy group. These data are very similar to those in the study by Cohen *et al*. (22), in which the immunotherapy drug is (pembrolizumab), obtaining a prevalence of OM of 6 cases (2%) compared to 28 cases (12%) in the standard therapy group. It should be noted that the study by Kiyota *et al*. (23) which reflects the Asian population data from the Ferris *et al*. (21) Nivolumab trial, mentioned above, describes a prevalence of OM of 1 case (4.3%) in the nivolumab group versus 3 cases (27.3%) in the standard therapy group.

The other three trials included in this review do not have a standard therapy group to establish a comparison, although it should be noted that the data obtained about the prevalence of OM are very similar to those obtained in phase III clinical trials. The study by Tahara *et al*. (24) shows a prevalence of OM of 1 case (4% of cases), in the study by Chow *et al*. (25), OM occurs in 2 cases (1% of cases) and in the study by Mehra *et al*. (26) a total of 4 cases were registered (2%).

The quality of the clinical trials analyzed in this review is high, even though in three of six articles, there is no group to establish a comparison, therefore these studies already mentioned, did not present a random allocation.

- Limitations

The limitations present in this review are mainly related to the presence of early phase studies and therefore, with samples of a disparate number compared to more advanced trials, as well as the lack of description by these studies of the grading of OM according to the WHO (The clinical trials included have used the National Cancer Institute Common Terminology Criteria for Adverse Events). This creates a lack of information in this regard but despite these limitations, it is possible to extract from this review that the prevalence of OM in new oral cancer immunotherapy drugs is significantly lower than in standard therapy used to date.

Despite being an article excluded from the review because it did not meet the inclusion criteria, the analysis of quality of life performed by Harrington *et al*. [2017] should be highlighted (27). Using a questionnaire (EORTC QLQ-H&N35) from the European Organisation for the Research and Treatment of Cancer (EORTC) in the CheckMate 141 clinical trial, they obtained results such as that the nivolumab group suffered a statistically significant deterioration in a more extensive period in comparison to the standard therapy group and furthermore, as for the study of pain, it was significantly lower in the nivolumab group. Some characteristics that were also studied at the oral level (opening problems, teeth problems, coughing, use of analgesics, weight loss, dry mouth, and feeling of discomfort) occurred later in the immunotherapy group (27).

Despite this, oral signs and symptoms of OM were not defined, however, the description of signs and symptoms made by the authors, can be related to a mucositis grade 3 according to the WHO classification (27).

## Conclusions

The prevalence of OM is lower in the new immunotherapy with monoclonal antibodies against oral cancer compared to the drugs used so far (chemotherapeutics [methotrexate, cisplatin] as well as cetuximab). However, further studies should be carried out to confirm these data.
